# Chronic Obstructive Pulmonary Disease Overdiagnosis and Overtreatment: A Meta-Analysis

**DOI:** 10.3390/jcm12226978

**Published:** 2023-11-08

**Authors:** Matteo Fiore, Matteo Ricci, Annalisa Rosso, Maria Elena Flacco, Lamberto Manzoli

**Affiliations:** 1 Section of Hygiene and Preventive Medicine, University of Bologna, 40126 Bologna, Italy; matteo.fiore7@studio.unibo.it (M.F.); matteo.ricci18@studio.unibo.it (M.R.); 2 Department of Environmental and Prevention Sciences, University of Ferrara, 44121 Ferrara, Italy; annalisa.rosso@unife.it (A.R.); mariaelena.flacco@unife.it (M.E.F.)

**Keywords:** chronic obstructive pulmonary disease (COPD), overdiagnosis, overtreatment, meta-analysis

## Abstract

This meta-analysis of observational studies aimed at estimating the overall prevalence of overdiagnosis and overtreatment in subjects with a clinical diagnosis of Chronic Obstructive Pulmonary Disease (COPD). MedLine, Scopus, Embase and Cochrane databases were searched, and random-effect meta-analyses of proportions were stratified by spirometry criteria (Global Initiative for COPD (GOLD) or Lower Limit of Normal (LLN)), and setting (hospital or primary care). Forty-two studies were included. Combining the data from 39 datasets, including a total of 23,765 subjects, the pooled prevalence of COPD overdiagnosis, according to the GOLD definition, was 42.0% (95% Confidence Interval (CI): 37.3–46.8%). The pooled prevalence according to the LLN definition was 48.2% (40.6–55.9%). The overdiagnosis rate was higher in primary care than in hospital settings. Fourteen studies, including a total of 8183 individuals, were included in the meta-analysis estimating the prevalence of COPD overtreatment. The pooled rates of overtreatment according to GOLD and LLN definitions were 57.1% (40.9–72.6%) and 36.3% (17.8–57.2%), respectively. When spirometry is not used, a large proportion of patients are erroneously diagnosed with COPD. Approximately half of them are also incorrectly treated, with potential adverse effects and a massive inefficiency of resources allocation. Strategies to increase the compliance to current guidelines on COPD diagnosis are urgently needed.

## 1. Introduction

According to the Global Burden of Disease, in 2019, Chronic Obstructive Pulmonary Diseases (COPDs) were the third most common cause of death across the world, causing over 3.3 million deaths [1]. In addition, the current literature consistently predicts a substantial increase in the future health burden of COPD [2]. Despite clear criteria for the diagnosis of COPD, produced by the Global Initiative for Chronic Obstructive Lung Disease (GOLD), being available for two decades [3,4], underdiagnosis and overdiagnosis are still common, causing, in turn, under- or overtreatment, and determining a suboptimal disease management [5,6,7,8]. One recent meta-analysis quantified the rate of underdiagnosis in primary healthcare [9], and several studies estimated the rate of overdiagnosis [10]. However, the available evidence is highly heterogeneous, and a summary estimate of the magnitude of overdiagnosis is not yet available. We thus carried out a systematic review and meta-analysis to estimate the overall prevalence of overdiagnosis in subjects with a clinical diagnosis of COPD, both in primary care and hospital settings.

## 2. Materials and Methods

### 2.1. Search Strategy and Data Extraction

The reporting of this systematic review was guided by the standards of the Preferred Reporting Items for Systematic Review and Meta-Analysis (PRISMA) 2020 Statement [11]. We extracted data from observational studies evaluating the false positive rate of clinical diagnosis (CD) compared to the spirometry confirmation. We searched MedLine, Scopus, Embase and Cochrane databases, up to 30 March 2023, using the following search strategy: “(COPD) AND (Misdiagnosis OR Overdiagnosis OR Overtreatment)” with the filter for years from 1997–2023. The time frame was chosen according to the theorization and introduction of the Global Initiative for Chronic Obstructive Lung Disease (GOLD) guidelines [12]. The references of the reviews and retrieved articles were also screened for additional pertinent papers. Only English-language studies were included. The extended version of the string is available in Table S1.

Each included article was independently evaluated by two reviewers (MF, and MR), who extracted the main study characteristics (first author, publication year, country, study design, population, setting, mean age of the CD-COPD patients, COPD definition, number of false positives, number of patients with CD and overtreated patients among overdiagnosed). Each of the reviewers extracted the data of the same set of articles by using an extraction table. Disagreements were discussed with and solved by a third reviewer (LM).

### 2.2. Data Analysis

According to the International GOLD guidelines [4] on the management of COPD, all clinical diagnoses must be confirmed by spirometry testing showing the airway’s irreversible obstructions. The primary outcome was the rate of overdiagnosis, defined as the number of subjects with a clinical diagnosis of COPD that was not confirmed after spirometry, divided by all the subjects with a clinical diagnosis of COPD (either confirmed or not after spirometry).

COPD clinical diagnosis was defined by the presence of one of the following [10]:

- Clinical diagnosis by a physician during the study or in recorded administrative data;
- History of medication coherent to COPD diagnosis;
- Physician ignoring a negative result of the spirometry.

A clinical diagnosis of COPD was considered appropriate when confirmed by spirometry using GOLD [4] and/or LLN (Lower Limit of Normal) [13] criteria. According to the GOLD definition, a diagnosis of COPD is confirmed when the spirometry shows a post-bronchodilator FEV1/FVC < 0.7 [4]. According to LLN criterion, a COPD diagnosis is confirmed when the spirometry shows a FEV1/FVC ratio that falls outside of two standard deviations of a reference population [13].

The secondary outcome was the rate of overtreatment among overdiagnosed subjects. It was defined as the number of subjects undergoing at least one COPD therapy, divided by all the overdiagnosed subjects. We used random-effect meta-analyses of proportions to combine data and obtain summary estimates of each outcome. The effect sizes (% of overdiagnosis or overtreatment, ES) and 95% Confidence Interval (CI) of each individual study were displayed using forest plots, in which studies’ ESs are graphically represented by dots, and their CIs are expressed as horizontal bars. All analyses were stratified by setting of care—hospital/healthcare center and primary care/general population—and were carried out using Stata, version 15.0 (Stata Corp., College Station, TX, USA, 2022).

### 2.3. Quality Assessment

The internal quality of each included report was assessed using the checklist recommended by The Strengthening the Reporting of Observational Studies in Epidemiology (STROBE) statement [14], composed of 22 items to evaluate the quality of observational study reports. STROBE does not provide ways to define a score allowing to rate the quality of the study. To investigate the potential impact of study quality in stratified meta-analyses, studies were classified as of “poor quality” if their overall score ranged from 0 to 14, of “intermediate quality” when the scores ranged from 15 to 25, of “good quality” when the scores were higher than 26 [15].

## 3. Results

### 3.1. Characteristics of the Included Studies

**Figure 1:**
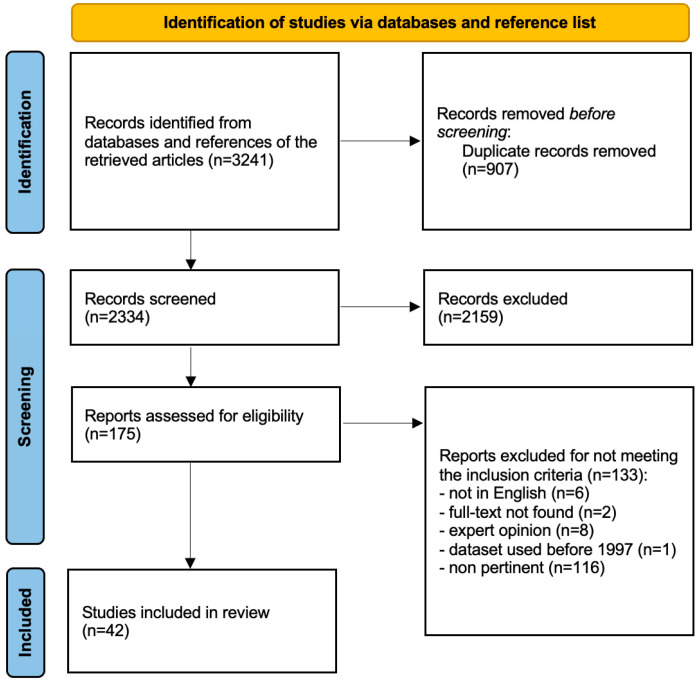
PRISMA flowchart. From: Page MJ, McKenzie JE, Bossuyt PM, et al. “The PRISMA 2020 statement: an updated guideline for reporting systematic reviews” [11].

The initial search identified 3241 articles; 907 articles were removed because duplicates, and 2159 were excluded at the title/abstract screening stage. The remaining 175 full-text articles were assessed for eligibility, and 42 papers met the criteria for final inclusion [8,16,17,18,19,20,21,22,23,24,25,26,27,28,29,30,31,32,33,34,35,36,37,38,39,40,41,42,43,44,45,46,47,48,49,50,51,52,53,54,55,56], (Figure 1).

The main characteristics of the included studies are reported in Table S2: these were published from 2005 to 2022; 20 were carried out in Europe [8,16,17,19,22,23,26,28,31,35,36,37,38,42,45,47,48,50,51,55], twelve in America [18,20,24,25,27,29,30,33,41,44,49,52], four in Oceania [34,40,54,56], two in Asia [21,39] and four were multicentric [32,43,46,53]. Almost all studies included had a cross-sectional design [8,16,17,18,19,20,21,22,23,25,27,28,29,31,32,33,34,35,36,37,38,39,40,41,42,43,44,45,46,47,48,49,50,51,52,53,54,55,56], and the cross-sectional data were extracted from three cohort studies [24,26,30]. In the 42 included papers, we were able to extract 33 datasets that recruited the participants from the general population or primary care patients [8,17,18,21,23,24,25,26,27,28,29,31,32,33,34,35,36,38,39,40,41,42,45,46,47,48,50,51,52,53,54,55,56], six that were performed in hospitals [19,20,30,37,44,49], and three datasets that recruited participants from both settings [16,22,43]. Thirty-two datasets used GOLD criteria to define COPD [8,16,17,18,21,23,24,25,26,27,28,29,30,31,33,34,35,36,38,39,41,42,45,47,48,50,51,52,53,54,55,56], three used LLN definition [19,20,32], and seven datasets adopted both definitions [22,37,40,43,44,46,49].

### 3.2. Quality Assessment

As reported in Table S3, according to the STROBE checklist, nine studies were classified as of “good quality”, and the remaining 33 as of “intermediate quality”. The most frequent issues pertained the description of potential biases in the Methods (incomplete in all but two studies), the explanation of the statistical analyses (unsatisfactory in all but eight studies), and the indication of the study design with commonly terms in the title or abstract (incomplete in all but eleven studies).

### 3.3. Overdiagnosis

**Figure 2:**
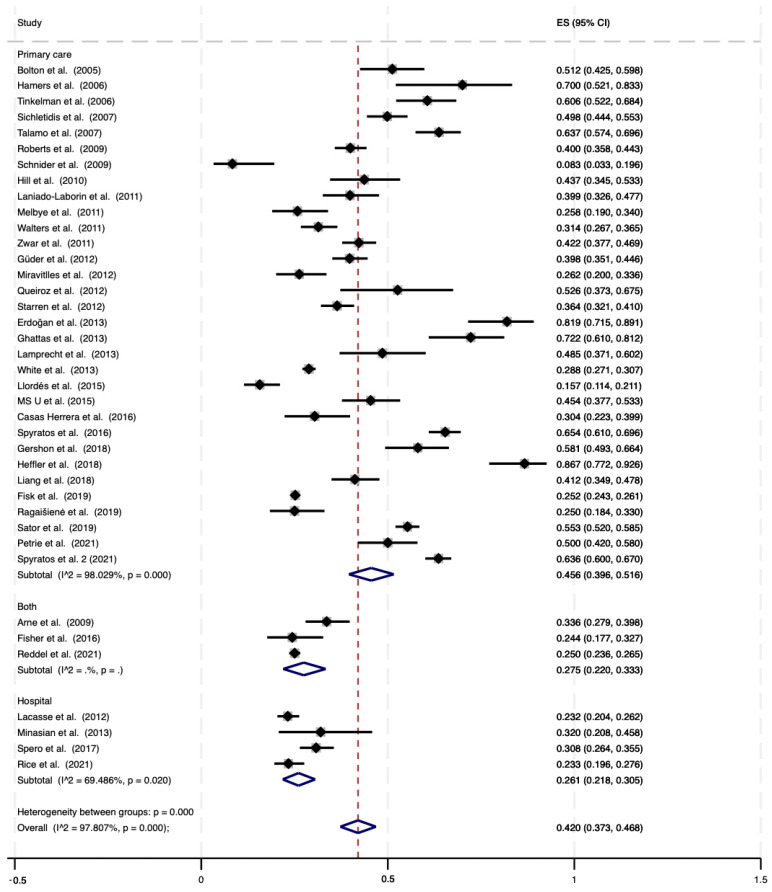
Proportion meta-analysis of overdiagnosis among subjects with clinical diagnosis of COPD, according to GOLD definition [8,16,17,18,21,22,23,24,25,26,27,28,29,30,31,33,34,35,36,37,38,39,40,41,42,43,44,45,46,47,48,49,50,51,52,53,54,55,56]. ES = Effect Size (% of overdiagnosis); CI: Confidence Interval; COPD: Clinically Diagnosed Chronic Obstructive Pulmonary Disease; GOLD: Global Initiative for Chronic Obstructive Lung Disease; I^2^: level of heterogeneity.

Thirty-nine datasets, including a total of 23,765 subjects, were included in the meta-analysis estimating the prevalence of COPD overdiagnosis according to GOLD definition (Table 1). Overall, the pooled prevalence was 42.0% (95% Confidence Interval (CI): 37.3–46.8% Figure 2), with a large heterogeneity among the individual studies. Five datasets showed a prevalence lower than 25%, while thirteen studies reported values higher than 50%. The summary prevalence of COPD overdiagnosis was significantly higher in the 32 primary care studies (45.6%; 95% CI: 39.6–51.6%) rather than in the four studies that included patients in hospital setting (26.1%; 95% CI: 21.8–30.5%). Among the latter four studies, three included only inpatients [30,44,49] and one included only outpatients [37]. The summary rate of overdiagnosis of the three studies including only inpatients was 25.5% (95% CI: 21.1–30.2%; Table 1).

**Figure 3:**
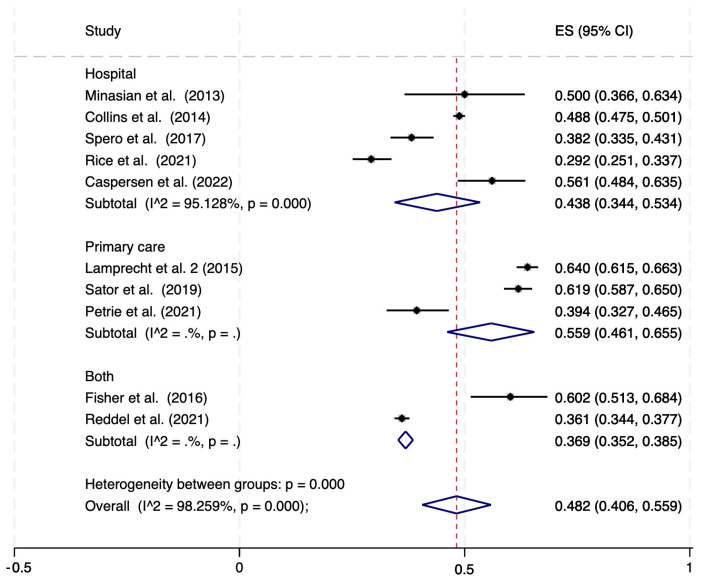
Proportion meta-analysis of overdiagnosis among subjects with clinical diagnosis of COPD, according to LLN definition [19,20,22,32,37,40,43,44,46,49]. ES = Effect Size (% of overdiagnosis); CI: Confidence Interval; COPD: Clinically Diagnosed Chronic Obstructive Pulmonary Disease; LLN: Lower limit of normal; I^2^: level of heterogeneity.

Ten studies quantified the proportion of COPD overdiagnosis according to LLN definition and included a total of 12,455 subjects (Table 1). Overall, the pooled prevalence was 48.2% (95% CI: 40.6–55.9%) (Figure 3), with the two studies including the primary care setting reporting values higher than 60%, and the five hospital-based studies showing a summary prevalence of 43.8% (95% CI: 34.4–53.4%—Figure 3). When the analyses were restricted to the seven studies that evaluated COPD prevalence using both GOLD and LLN criteria, on the same population, the pooled prevalence of COPD according to GOLD and LLN criteria were, respectively, 34.0% (95% CI: 23.0–46.0%) and 47.5% (95% CI: 36.5–58.5%) (Figure S1).

### 3.4. Overtreatment

**Figure 4:**
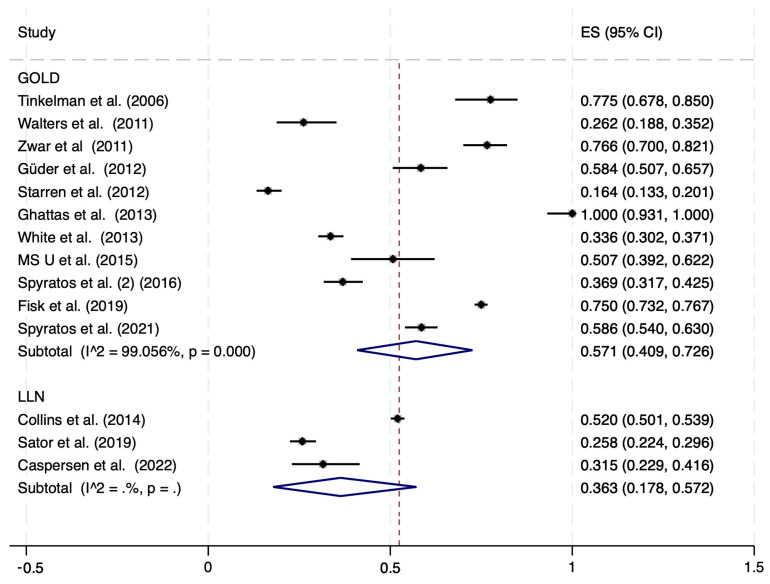
Proportion meta-analysis of overtreatment among overdiagnosed individuals, according to GOLD and LLN definition [8,19,20,23,25,26,39,46,50,51,53,54,55,56]. ES = Effect Size (% of overtreatment); CI: Confidence Interval; COPD: Clinically Diagnosed Chronic Obstructive Pulmonary Disease; GOLD: Global Initiative for Chronic Obstructive Lung Disease; LLN: Lower limit of normal; I^2^: level of heterogeneity.

Eleven studies, including a total of 4842 individuals, were included in the meta-analysis estimating the prevalence of COPD overtreatment according to GOLD definition (Table S2). Four studies reported a proportion of overtreated subjects, among those that were overdiagnosed, equal or larger than 75% (Figure 4), but the overall estimated prevalence was 57.1% (95% CI: 40.9–72.6%). When the results of the three studies (3341 subjects) that evaluated the proportion of overtreatment according to LLN definition were pooled, the summary prevalence was 36.3% (95% CI: 17.8–57.2%; Figure 4).

## 4. Discussion

The main findings of this meta-analysis, which included the data of more than 24,000 subjects with a clinical diagnosis of COPD from 35 different countries, are the following: (a) when spirometry was not used, at least four patients out of ten received an erroneous diagnosis of COPD, with rates substantially higher in primary care; (b) the prevalence of overdiagnosis did not decrease over time, and was still higher than 50% in three recent studies [19,46,50]; (c) more than half of the overdiagnosed subjects received an inappropriate COPD treatment; (d) on the same population of subjects, the prevalence of COPD overdiagnosis was consistently lower when GOLD rather than LLN criteria were used.

The false positive prevalence may be attributed to the physician’s incapacity of distinguishing COPD to other clinical conditions due to the similar symptomatology with other diseases [57,58] and the under-use of spirometry [28]. Patients with overlapping COPD symptoms, like cough, breathlessness and dyspnea, may be empirically labeled by the physician as “GOLD 0”, leading to possible diagnostic confusion [59,60]. Conditions like asthma, obesity, cardiac pathologies, restrictive patterns, and aging may be the condition underneath the refereed symptoms [61,62]. Further studies are necessary to explore the prevalence of the underlying condition in the case of false positive CD-COPD. These epidemiological insights may help physicians to make appropriate clinical decisions and policy makers to design better diagnostic-therapeutic pathways.

The use of spirometry testing is highlighted by guidelines to prevent overdiagnosis; still, there are no signs of a substantial improvement in its compliance by the clinicians in recent years. The under-use of spirometry testing may be explained by barriers that span in multiple domains. Ranging from a lack of awareness regarding the importance of assessing lung function [63], to difficulties in accessing spirometry evaluation [64,65], to issues concerning the interpretation of spirometry patterns by primary care physicians [66]. The burden of overdiagnosis is higher in the primary care setting with respect to the hospital. The difference may be driven by the hospital setting having less barriers to guideline implementations [67]. However, further studies are needed to clarify the reason underneath this difference [68]. Considering these challenges, policy makers should welcome all Public Health strategies to improve guideline adherence. Reasonable approaches could be: (a) education of General Practitioners (GPs) to use a well-funded wait-see approach [28]; (b) increase the patient’s awareness about the “too much medicine problem” [69]; (c) creation of spirometry specialized hubs in a coordinate Primary Care network [70]; (d) GPs equipped and trained for the spirometry use [71]; (e) restrictive rules in the drugs prescription [72].

Overall, approximately half of the 40% subjects that were overdiagnosed received an inappropriate COPD treatment, which translates in approximately one patient out of five with a suspect COPD being overtreated. Indeed, overtreatment is unlikely to produce a net benefit for the patients [73], potentially leading to several adverse effects, from cough to pneumonia, and to a delayed diagnosis of the true condition that caused the respiratory symptoms [55]. In addition, as estimated by some global analyses, the costs associated with overtreatment can be massive [74,75,76,77]. The global therapeutics market size of COPD is estimated at $20 billion in 2023 and is projected to reach approximately $33 billion by 2030 [78]. Thus, according to the present findings, every year billions of USD may be potentially wasted on overtreatment for “GOLD 0” patients. Moreover, as White et al. suggested, the overtreatment may not be limited to GOLD 0, but may extend to GOLD 1, 2, 3, and 4 [55].

In this scenario, it may be reassuring that, at least in theory, the solution is relatively straightforward, as an adequate use of spirometry ensures accurate diagnosis and treatment and reduces unnecessary treatment [27,51]. According to Spyratos et al., the resources saved thanks to a proper spirometry-based diagnosis could potentially cover the entire cost of treatment for the underdiagnosed population, thus diverting funds from more urgent and important illnesses needs [8]. Although further studies are needed to more precisely assess the financial upsides of diagnostic adherence to the guidelines, the results of this meta-analysis strongly reinforce the call for strategies that may substantially increase the adherence to current COPD guidelines in all settings.

The American Thoracic Society and the European Respiratory Society recommend the use of age- and sex-specific LLN definition for FEV1/FVC, which may lead to a more precise COPD assessment [79]. However, the meta-analyses stratified by COPD definition showed a higher rate of overdiagnosis when LLN was used. Moreover, in all of the seven studies that estimated the rate of overdiagnosis using both LLN and GOLD definitions, on the same subjects [22,37,40,43,44,46,49], the raw proportion of overdiagnosed subjects was higher when LLN criteria were adopted, and the overall prevalence of overdiagnosis was 34.0% using GOLD definition; 47.5% using LLN. On the other side, however, the proportion of overtreated subjects was higher when GOLD criteria were used (57.1% vs. 36.3% using LLN criteria). While a higher rate of overdiagnosis might be expected when LLN is used, since the GOLD “fixed ratio” approach is known to overestimate COPD in older individuals (given the progressive FEV1/FVC ratio decrease with age) [80], being thus being less efficient at recognizing the errors by the clinicians, the lower proportion of overtreatment that was observed using LLN criteria was unexpected and may be due, at least in part, by the sum of a statistical and an epidemiological issue. Firstly, only three studies were included in the meta-analysis estimating LLN overtreatment, and the summary rate was heavily influenced by the smaller samples, with the arithmetic mean being substantially larger (47.0%) than the weighted one (36.3%). Secondly, and more importantly, while all the studies that evaluated overtreatment using GOLD criteria were performed in primary care (which was associated with higher rates of overtreament), two of the three studies (and 83% of the total sample) that adopted LLN criteria were carried out in the hospital setting, where lower overtreatment rates were observed). Although the above factors may partially explain the difference in overtreatment prevalence that was observed adopting GOLD or LLN definitions, the overall findings provide support to the intense controversy over which criteria to use for the spirometry definition of COPD [81]. Indeed, reaching a consensus on this point is both urgent and essential to proceed with uniform and widely accepted strategies to reduce both overdiagnosis and overtreatment [82].

This meta-analysis has some limitations that must be considered in interpreting the results. First, as with any systematic review, publication bias may have influenced the results. However, this was a meta-analysis of proportions, with no direct comparisons, thus avoiding the typical bias deriving from the lower publication rate of non-significant results [83]. Second, the retrospective studies dealing with hospital data are at high risk of misclassification bias. Notably, however, the results of these studies showed a lower prevalence of COPD overdiagnosis. Third, the number of datasets evaluating overdiagnosis or overtreatment according to LLN definition was limited, although the overall sample was larger than 3000 patients for both outcomes. Fourth, we extracted spirometry-based estimates using the LLN and GOLD criteria, but these have some limitations. Different studies may use distinct tools, spirometers, and protocols, which may contribute to the high heterogeneity in the prevalence rates. Unfortunately, other potential definitions of overdiagnosis, such as normalization after therapy with multiple spirometry follow-ups [35], or use of a pre-bronchodilator, were adopted in very few studies [84,85] and could not be explored with a meta-analysis [10]. Fifth, most meta-analyses showed a high level of heterogeneity. However, of the 42 studies that were included in the meta-analysis to estimate the degree of overdiagnosis, 37 reported a prevalence of overdiagnosis higher than 25%; of the 14 studies included to estimate the overtreatment, eleven reported a prevalence higher than 30%. Thus, although a precise estimate cannot be obtained, it is very unlikely that the mean incidence of overdiagnosis and overtreatment are actually lower than 25% and 30%. Lastly, we only searched for studies written in English, which may have caused a selection bias, since other regions show greater difficulties in adhering to the recommendations and management guidelines for COPD [86], which in turn causes greater overdiagnosis of hospitalized patients and underdiagnosis in relation to the general population.

## 5. Conclusions

This meta-analysis shows that, when the diagnosis of COPD is exclusively clinical, and no spirometry is used, four out of ten patients are erroneously diagnosed with COPD, and should be further examined to ascertain the true causes of the respiratory symptoms. The proportion of overdiagnosis was substantially higher in the primary setting. In addition, approximately half of the overdiagnosed subjects are erroneously treated for COPD, with potential adverse effects and a massive inefficiency of resources allocation. The prevalence of overdiagnosis did not decrease over time and was higher when LLN rather than GOLD definition was adopted. These findings strongly reinforce the need to increase compliance to current guidelines on the use of spirometry in COPD diagnostic process.

## Figures and Tables

**Table 1:** Pooled rates of COPD overdiagnosis and overtreatment.

Outcome: Overdiagnosis	Study Ref.	N. Datasets (n/N) ^a^	Pooled Rates % (95% CI)	I^2^, %
COPD Definition: GOLD				
Overall sample		39 (7710/23,765)	42.0 (37.3–46.8)	97.8%
Primary care/ general population setting	[8,17,18,21,23,24,25,26,27,28,29,31,33,34,35,36,38,39,40,41,42,45,46,47,48,50,51,52,53,54,55,56]	32 (6356/18,450)	45.6 (39.6–51.6)	98.0%
Hospital/ healthcare center setting	[30,37,44,49]	4 (421/1666)	26.1 (21.8–30.5)	69.5%
Hospital/inpatients	[30,44,49]	3 (405/1616)	25.5(21.1–30.2)	--
Hospital/outpatients	[37]	1 (16/50)	32.0 (19.5–46.7)	--
Both settings	[16,22,43]	3 (933/3649)	27.5 (22.0–33.3)	--
COPD Definition: LLN				
Overall sample		10 (5917/12,455)	48.2 (40.6–55.9)	98.3%
Primary care/ general population setting	[32,40,46]	3 (1619/2611)	55.9 (46.1–65.5)	--
Hospital/ healthcare center setting	[19,20,37,44,49]	5 (3070/6521)	43.8 (34.4–53.4)	95.1%
Both settings	[22,43]	2 (1228/3323)	36.9 (35.2–38.5)	--
**Outcome: Overtreatment**	**Study ref.**	**N. datasets** **(n/N) ^b^**	**Pooled rates** **% (95% CI)**	**I^2^, %**
COPD Definition: GOLD	[8,23,25,26,39,50,51,53,54,55,56]	11 (2807/4842)	57.1 (40.9–72.6)	99.1%
COPD Definition: LLN	[19,20,46]	3 (1570/3341)	36.3 (17.8–57.2)	98.6%

^a^ Number of patients overdiagnosed among number of CD-COPD patients. ^b^ Number of patients overtreated among total number of patients overdiagnosed. CD-COPD: Clinically Diagnosed-Chronic Obstructive Pulmonary Disease. GOLD: Global Initiative for Chronic Obstructive Lung Disease. LLN: Lower limit of normal. CI: Confidence Interval. I^2^: level of heterogeneity.

## Data Availability

All data are available upon request from the corresponding author.

## Supplementary Materials

The following supporting information can be downloaded at: https://www.mdpi.com/article/10.3390/jcm12226978/s1, Table S1: Search strategies’ extended version; Table S2: Characteristics and main results of the studies included in the meta-analysis on overdiagnosis; Table S3: Quality assessment of observational studies. The Strengthening the Reporting of Observational Studies in Epidemiology (STROBE) statement; Figure S1: Prevalence of overdiagnosed in the same samples, according to GOLD and LLN definition; Figure S2: Proportion meta-analysis of overdiagnosis among Outpatients (OUT) and Inpatients (IN), according to GOLD definition.
