# Supplementary material for: Chronic Obstructive Pulmonary Disease Overdiagnosis and Overtreatment: A Meta-Analysis
Source: J Clin Med. 2023 Nov 8;12(22):6978. doi: 10.3390/jcm12226978 (PMC10672453; doi:10.3390/jcm12226978)
Supplement: Supplementary file 1 [file jcm-12-06978-s001.zip › jcm-2617232-supplementary.pdf]

Table S1. Search strategies' extended version.

**PUBMED**	(("pulmonary disease, chronic obstructive"[MeSH Terms] OR ("pulmonary"[All Fields] AND "disease"[All Fields] AND "chronic"[All Fields] AND "obstructive"[All Fields]) OR "chronic obstructive pulmonary disease"[All Fields] OR "copd"[All Fields])) AND (("diagnostic errors"[MeSH Terms] OR ("diagnostic"[All Fields] AND "errors"[All Fields]) OR "diagnostic errors"[All Fields] OR "misdiagnoses"[All Fields] OR "misdiagnosis"[All Fields] OR "misdiagnose"[All Fields] OR "misdiagnosed"[All Fields] OR "misdiagnosing"[All Fields]) OR "overdiagnosis"[MeSH Terms] OR "overdiagnosis"[All Fields])) AND ("hasabstract"[All Fields] AND 1997/01/01:2023/03/30[Date - Publication])
**SCOPUS**	(copd OR chronic AND obstructive AND pulmonary AND disease) AND (misdiagnosis OR overdiagnosis OR wrong AND diagnosis OR erroneous AND diagnosis OR overtreatment OR wrong AND treatment OR erroneous AND treatment) AND PUBYEAR > 1996 AND PUBYEAR < 2024 AND (LIMIT-TO (SUBJAREA , "MEDI")) AND (LIMIT-TO (DOCTYPE , "ar") OR LIMIT-TO (DOCTYPE , "re")) AND (LIMIT-TO (PUBSTAGE , "final")) AND (LIMIT-TO (SRCTYPE , "j")) AND (LIMIT-TO (LANGUAGE , "English"))
**EMBASE**	(COPD) AND (Misdiagnosis OR Overdiagnosis OR Overtreatment)
**COCHRANE**	(copd OR chronic AND obstructive AND pulmonary AND disease) AND (misdiagnosis OR overdiagnosis OR wrong AND diagnosis OR erroneous AND diagnosis OR overtreatment OR wrong AND treatment OR erroneous AND treatment)

Table S2. Characteristics and main results of the included studies.

First Author	Year	Country	Study Design	Population Characteristics; Age selection	CD-COPD Mean Age (SD)	Setting	Definition A.O.	n/N[a]	Total overtreated/ total overdiagnosed	Notes
Bolton et al. [17]	2005	United Kingdom	Cross-sectional study*	Patients with COPD diagnosis without spirometry; Age NA	64.3 (NA)	Primary care/ general population	Post-bronchodilator FEV1/FVC < 0.7 (GOLD)	64/125		
Hamers et al. [27]	2006	Brazil	Cross-sectional study*	Patients attended the GP with symptoms of shortness of breath and/or cough, irrespective the cause or clinical history; Age≥ 15	46.8 (18.7)**	Primary care/ general population	Post-bronchodilator FEV1/FVC < 0.7 (GOLD)	21/30		Mean age computed for 350 subjects
Tinkelman et al. [53]	2006	Multiple Countries (United Kingdom and United States)	Cross-sectional study*	Subjects in general practices lists with prior diagnoses or medications coherent with COPD; Age≥ 40	58.7 (11.4)**	Primary care/ general population	Post-bronchodilator FEV1/FVC < 0.7 (GOLD)	83/137	69/89	Mean age computed for 597 subjects
Sichletidis et al. [48]	2007	Greece	Cross-sectional study*	Patients with COPD diagnosis under medication; Age>40	NA (NA)	Primary care/ general population	Post-bronchodilator FEV1/FVC < 0.7 (GOLD)	159/319		
Tálamo et al. [52]	2007	Multiple Countries (Latin American Countries)	Cross-sectional study*	Randomly selected; Age ≥40	55.6 (11.0)^	Primary care/ general population	Post-bronchodilator FEV1/FVC < 0.7 (GOLD)	151/237		^Mean age computed for 151 subjects (only false positive subjects)

Arne et al. [16]	2009	Sweden	Cross-sectional study	Randomly selected COPD patients, with recent diagnosis; Age 18-75	59.2 (8.3)**	Both settings	Post-bronchodilator FEV1/FVC < 0.7 (GOLD)	81/241		Mean age computed for 316 subjects
Roberts et al. [45]	2009	United Kingdom	Cross-sectional study*	Patients with suspected COPD diagnosis, referrals for general hospital spirometry; Age NA	63.8 (11.3)**	Primary care/ general population	Post-bronchodilator FEV1/FVC < 0.7 (GOLD)	201/503°		Mean age computed for 503 subjects. °Both, suspected (78/177) or definite (123/326) COPD diagnosis
Roberts et al. [45]	2009	United Kingdom	Cross-sectional study*	Patients with suspected COPD diagnosis, referrals for general hospital spirometry; Age NA	63.8 (11.3)**	Primary care/ general population	Post-bronchodilator FEV1/FVC < 0.7 (GOLD)	78/177		Suspected diagnosis of COPD
Roberts et al. [45]	2009	United Kingdom	Cross-sectional study*	Patients with suspected COPD diagnosis, referrals for general hospital spirometry; Age NA	63.8 (11.3)**	Primary care/ general population	Post-bronchodilator FEV1/FVC < 0.7 (GOLD)	123/326		Definite diagnosis of COPD
Schneider et al. [47]	2009	Germany	Cross-sectional study	Patients with respiratory symptoms; Age NA (adults)	43.8 (15.6)**	Primary care/ general population	Post-bronchodilator FEV1/FVC < 0.7 (GOLD)	4/48		Mean age computed for 219 subjects
Hill et al. [29]	2010	Canada	Cross-sectional study*	Having a ≥ 20 pack-year history of smoking; Age>40	65.6 (9.3)	Primary care/ general population	Post-bronchodilator FEV1/FVC < 0.7 (GOLD)	45/103		
Laniado-Laborin et al. [33]	2011	Mexico	Cross-sectional study*	Patients with known risk factors referrals for spirometry; Age≥40	57.6 (12.2)**	Primary care/ general population	Post-bronchodilator FEV1/FVC < 0.7 (GOLD)	63/158		Mean age computed for 2293 subjects
Melbye et al. [36]	2011	Norway	Cross-sectional study*	COPD or asthmatic patients; Age≥40	66.7 (NA)**	Primary care/ general population	Post-bronchodilator FEV1/FVC < 0.7 (GOLD)	33/128		Mean age computed for 166 subjects
Zwar et al. [56]	2011	Australia	Cross-sectional study	Patients with COPD drug prescription;	65.6 (10.1)	Primary care/ general	Post-bronchodilator FEV1/FVC < 0.7	188/445	144/188	

				Age 40-80		population	(GOLD)			
Walters et al. [54]	2011	Australia	Cross-sectional study	CD-COPD (smokers or pack-year smoking history≥10 years); Age NA	62.6 (8.1)	Primary care/ general population	Post-bronchodilator FEV1/FVC < 0.7 (GOLD)	107/341	28/107***	
Güder et al. [26]	2012	Netherlands	Prospective cohort study	Patients with a GP's diagnosis of COPD; Age≥65	73(5.3)	Primary care/ general population	Post-bronchodilator FEV1/FVC < 0.7 (GOLD)	161/405	94/161***	
Lacasse et al. [30]	2012	Canada	Cohort study	Patients with COPD diagnosis at hospital discharge; Age NA	73.1 (12.2)**	Hospital/ healthcare center	Post-bronchodilator FEV1/FVC < 0.7 (GOLD)	186/802		Mean age computed for 1221 subjects
Miravitlles et al. [38]	2012	Spain	Cross-sectional study	Patients with chronic respiratory symptoms; Age>40	68.9 (9.7)**	Primary care/ general population	Post-bronchodilator FEV1/FVC < 0.7 (GOLD)	42/160		Mean age computed for 210 subjects
Queiroz et al. [41]	2012	Brazil	Cross-sectional study	Patient at risk (smoking history or exposure to biomass); Age≥ 40	65.0 (10.4)**	Primary care/ general population	Post-bronchodilator FEV1/FVC < 0.7 (GOLD)	20/38		Mean age computed for 200 subjects
Starren et al. [51]	2012	United Kingdom	Cross-sectional study*	Patients with definite and suspected COPD diagnosis, 1st appointment referrals for spirometry; Age NA	66.6 (11.8)°	Primary care/ general population	Post-bronchodilator FEV1/FVC < 0.7 (GOLD)	162/445	73/445	°Both, suspected (35/180) or definite (127/265) COPD diagnosis
Starren et al. [51]	2012	United Kingdom	Cross-sectional study*	Patients with definite and suspected COPD diagnosis, 1st appointment referrals for spirometry; Age NA	--	Primary care/ general population	Post-bronchodilator FEV1/FVC < 0.7 (GOLD)	35/180		Suspected diagnosis of COPD
Starren et al. [51]	2012	United Kingdom	Cross-sectional study*	Patients with definite and suspected COPD diagnosis, 1st appointment referrals for spirometry; Age NA	--	Primary care/ general population	Post-bronchodilator FEV1/FVC < 0.7 (GOLD)	127/265		Definite diagnosis of COPD

Erdoğan et al. [21]	2013	Turkey	Cross-sectional study	Patients who were admitted to the Primary Care Center; Age >40	55.0 (11.0)**	Primary care/ general population	Post-bronchodilator FEV1/FVC < 0.7 (GOLD)	59/72		Mean age computed for 500 subjects
Ghattas et al. [25]	2013	United States	Cross-sectional study*	Uninsured patients; Age NA	52.9 (7.7)	Primary care/ general population	Post-bronchodilator FEV1/FVC < 0.7 (GOLD)	52/80°	52/52	80 patients referred (72 COPD diagnoses, 8 using inhalers)
Lamprecht et al. [31]	2013	Austria	Cross-sectional study*	Random sample of population; Age>40	57.7 (11.4)**	Primary care/ general population	Post-bronchodilator FEV1/FVC < 0.7 (GOLD)	33/68		Mean age computed for 1258 subjects
Minasian et al. [37]	2013	Netherlands	Cross-sectional study*	Patients with LVEF<40%; Age NA	69.0 (10.0)**	Hospital/ healthcare center	Post-bronchodilator FEV1/FVC < 0.7 (GOLD)	16/50		Mean age computed for 187 subjects
Minasian et al. [37]	2013	Netherlands	Cross-sectional study*	Patients with LVEF<40%; Age NA	69.0 (10.0)**	Hospital/ healthcare center	Post-bronchodilator FEV1/FVC < LLN	25/50		Mean age computed for 187 subjects
White et al. [55]	2013	United Kingdom	Cross-sectional study	General population; Age NA	69.8 (12.0)**	Primary care/ general population	Post-bronchodilator FEV1/FVC < 0.7 (GOLD)	709/2458	238/709	Mean age computed for 3537 subjects
Collins et al. [20]	2014	United States	Cross-sectional study*	US veterans with CD-COPD; Age NA	65.9 (11.1)	Hospital/ healthcare center	Post-bronchodilator FEV1/FVC < LLN	2680/5493	1394/2680	
Lamprecht et al. [32]	2015	Multiple Countries	Cross-sectional study*	Adults randomly selected from administrative areas worldwide; Age≥40 (27072 subjects) Age 40-80 (3802 subjects)	56.1 (11.3)**	Primary care/ general population	Post-bronchodilator FEV1/FVC < LLN	982/1544***		Mean age computed for 30874 subjects
Llordés et al. [35]	2015	Spain	Cross-sectional study*	Patients with history of smoking in their medical records; Age>45	59.9 (9.8)**	Primary care/ general population	Post-bronchodilator FEV1/FVC < 0.7 (GOLD)	34/217		Mean age computed for 1738 subjects
MS U et al. [39]	2015	China (Macau)	Cross-sectional study	Patients with the diagnostic code R97 (International Classification for Primary Care for	67.9 (10.0)	Primary care/ general population	Post-bronchodilator FEV1/FVC < 0.7 (GOLD)	69/152	35/69	

				COPD); Age 40-85						
Casas Herrera et al. [18]	2016	Multiple Countries (Latin American Countries)	Cross-sectional study	Patients with risk for COPD (smokers or biomass); Age>40	NA (NA)	Primary care/ general population	Post-bronchodilator FEV1/FVC < 0.7 (GOLD)	31/102		
Fisher et al. [22]	2016	United Kingdom	Cross-sectional study*	People living at home or in institutional care and regardless of their current health status; Population 1921 Birth	85.5 (0.4)**	Both	Post-bronchodilator FEV1/FVC < 0.7 (GOLD)	30/123		Mean age computed for 845 subjects
Fisher et al. [22]	2016	United Kingdom	Cross-sectional study*	People living at home or in institutional care and regardless of their current health status; Population 1921 Birth	85.5 (0.4)**	Both	Post-bronchodilator FEV1/FVC < LLN	74/123		Mean age computed for 845 subjects
Spyratos et al. [8]	2016	Greece	Cross-sectional study	Current or former smokers (at least 10 pack-years); Age>40	60.5 (13.4)**	Primary care/ general population	Post-bronchodilator FEV1/FVC < 0.7 (GOLD)	306/468	113/306	Mean age computed for 3200 patients
Spero et al. [49]	2017	United States	Cross-sectional study*	Patients admitted with COPD as leading cause; Age>18	69.3 (12.5)	Hospital/ healthcare center	Post-bronchodilator FEV1/FVC < 0.7 (GOLD)	120/390		
Spero et al. [49]	2017	United States	Cross-sectional study*	Patients admitted with COPD as leading cause; Age>18	69.3 (12.5)	Hospital/ healthcare center	Post-bronchodilator FEV1/FVC < LLN	149/390		
Gershon et al. [24]	2018	Canada	Longitudinal cohort study	Randomly selected adults. COLD study data; Age≥40	64.0 (10.4)	Primary care/ general population	Post-bronchodilator FEV1/FVC < 0.7 (GOLD)	72/124		

Heffler et al. [28]	2018	Italy	Cross-sectional study*	The first 300 patients consecutively sent by GPs to "Ricerca & Respiro ONLUS" to perform a spirometric evaluation; Age NA	67.9 (10.0)	Primary care/ general population	Post-bronchodilator FEV1/FVC < 0.7 (GOLD)	65/75		
Liang et al. [34]	2018	Australia	Cross-sectional study*	Patients visiting GP clinics, current or ex-smokers with a smoking history of at least 10 pack-years; Age≥40	67.1 (10.6)	Primary care/ general population	Post-bronchodilator FEV1/FVC < 0.7 (GOLD)	91/221		Mean age computed for 245 subjects (participants without spirometric results were included in the mean age calculation)
Fisk et al. [23]	2019	United Kingdom	Cross-sectional study*	Patients registered living with COPD; Age NA	70.8 (10.3)	Primary care/ general population	Post-bronchodilator FEV1/FVC < 0.7 (GOLD)	2255/8957	1691/2255***	
Ragaišienė et al. [42]	2019	Lithuania	Cross-sectional study*	Patients with COPD diagnosis; Age NA	67.0 (14.0)**	Primary care/ general population	Post-bronchodilator FEV1/FVC < 0.7 (GOLD)	33/132		Mean age computed for 228 subjects
Sator et al. [46]	2019	Multiple Countries	Cross-sectional study*	Non-institutionalized adults, population-based; Age NA	NA (NA)	Primary care/ general population	Post-bronchodilator FEV1/FVC < 0.7 (GOLD)	508/919		
Sator et al. [46]	2019	Multiple Countries	Cross-sectional study*	Non-institutionalized adults, population-based; Age NA	NA (NA)	Primary care/ general population	Post-bronchodilator FEV1/FVC < LLN	569/919	147/569	
Petrie et al. [40]	2021	Australia	Cross-sectional study	BOLD Australia, adults non-institutionalised; Age ≥40	58.9 (11.7)**	Primary care/ general population	Post-bronchodilator FEV1/FVC < 0.7 (GOLD)	74/148		Mean age computed for 3357 subjects
Petrie et al. [40]	2021	Australia	Cross-sectional study	BOLD Australia, adults non-institutionalised; Age ≥40	58.9 (11.7)**	Primary care/ general population	Post-bronchodilator FEV1/FVC < LLN	88/148		Mean age computed for 3357 subjects

Reddel et al. [43]	2021	Multiple Countries	Cross-sectional study	Patients with a physician-assigned/suspected diagnosis of COPD, asthma or both; Age≥18 (age ≥12 for 11 countries)	66.6 (9.6)	Both	Post-bronchodilator FEV1/FVC < 0.7 (GOLD)	822/3285		
Reddel et al. [43]	2021	Multiple Countries	Cross-sectional study	Patients with a physician-assigned/suspected diagnosis of COPD, asthma or both; Age≥18 (age ≥12 for 11 countries)	66.6 (9.6)	Both	Post-bronchodilator FEV1/FVC < LLN	1154/3200		
Rice et al. [44]	2021	United States	Cross-sectional study*	COPD patients discharge from hospital in 2018; Age NA	65.2 (11.6)	Hospital/ healthcare center	Post-bronchodilator FEV1/FVC < 0.7 (GOLD)	99/424		
Rice et al. [44]	2021	United States	Cross-sectional study*	COPD patients discharge from hospital in 2018; Age NA	65.2 (11.6)	Hospital/ healthcare center	Post-bronchodilator FEV1/FVC < LLN	124/424		
Spyratos et al. [50]	2021	Greece	Cross-sectional study*	General population current and former smokers (at least 10 pack-years); Age>40	58.2 (12.7)**	Primary care/ general population	Post-bronchodilator FEV1/FVC < 0.7 (GOLD)	461/725	270/461	Mean age computed for 5226 subjects
Caspersen et al. [19]	2022	Norway	Cross-sectional study	Residents of Akershus County born in 1950; Age 62-65	NA (NA)	Hospital/ healthcare center	Post-bronchodilator FEV1/FVC < LLN	92/164	29/92	

[a]: number of patients overdiagnosed among number of CD-COPD patients. * study design was not explicitly defined with common terms ** mean age calculated on study's general population. *** calculated from refereed percentage in the paper.
CD-COPD: Chronic Obstructive Pulmonary Disease. SD: Standard Deviation. NA: Not Available. GP: General Practitioner. GOLD: Global Initiative for Chronic Obstructive Lung Disease. LLN: Lower limit of normal. LVEF: Left Ventricular Ejection Fraction. FEV1/FVC: Modified Tiffeneau-Pinelli index.

Table S3. Quality assessment of observational studies. The Strengthening the Reporting of Observational Studies in Epidemiology (STROBE) Statement.

References	Quality	Score
Arne et al. [16]	Intermediate	25/33
Bolton et al. [17]	Intermediate	20/33
Casas Herrera et al. [18]	Intermediate	25/33
Caspersen et al. [19]	Good	28/33
Collins et al. [20]	Good	26/33
Erdoğan et al. [21]	Intermediate	19/33
Fisher et al. [22]	Good	27/33
Fisk et al. [23]	Intermediate	24/33
Gershon et al. [24]	Intermediate	25/33
Ghattas et al. [25]	Intermediate	20/33
Güder et al. [26]	Good	28/33
Hamers et al. [27]	Intermediate	24/33
Heffler et al. [28]	Intermediate	18/33
Hill et al. [29]	Intermediate	25/33
Lacasse et al. [30]	Intermediate	23/33
Lamprecht et al. 2013 [31]	Intermediate	22/33
Lamprecht et al. 2015 [32]	Intermediate	25/33
Laniado-Laborin et al. [33]	Intermediate	24/33
Liang et al. [34]	Good	26/33
Llordés et al. [35]	Intermediate	24/33
Melbye et al. [36]	Intermediate	23/33
Minasian et al. [37]	Intermediate	25/33
Miravitlles et al. [38]	Intermediate	25/33
MS U et al. [39]	Intermediate	24/33
Petrie et al. [40]	Good	27/33
Queiroz et al. [41]	Intermediate	21/33
Ragaišienė et al. [42]	Intermediate	20/33
Reddel et al. [43]	Intermediate	24/33
Rice et al. [44]	Intermediate	23/33
Roberts et al. [45]	Intermediate	18/33
Sator et al. [46]	Intermediate	24/33
Schneider et al. [47]	Good	27/33
Sichletidis et al. [48]	Intermediate	22/33
Spero et al. [49]	Intermediate	25/33
Spyratos et al 2016 [8]	Intermediate	21/33
Spyratos et al. 2021 [50]	Intermediate	23/33
Starren et al. [51]	Intermediate	24/33
Tálamo et al. [52]	Intermediate	24/33
Tinkelman et al. [53]	Intermediate	21/33
Walters et al. [54]	Good	27/33
White et al. [55]	Good	28/33
Zwar et al. [56]	Intermediate	24/33

Figure S1. Prevalence of overdiagnosed in the same samples, according to GOLD and LLN definition.

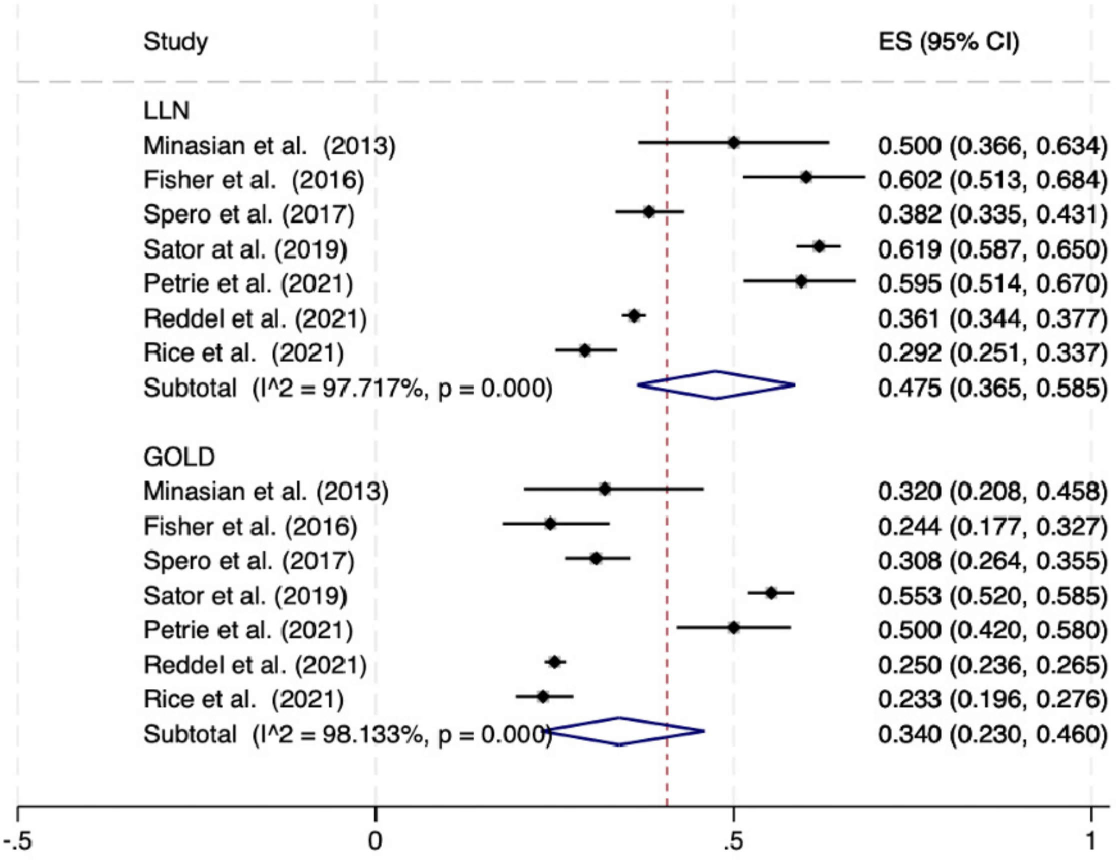

COPD: Clinical diagnosed Chronic Obstructive Pulmonary Disease. GOLD: Global Initiative for Chronic Obstructive Lung Disease. LLN: Lower limit of normal. CI: Confidence Interval. I^2: level of heterogeneity.

Figure S2. Proportion meta-analysis of overdiagnosis among Outpatients (OUT) and Inpatients (IN), according to GOLD definition.

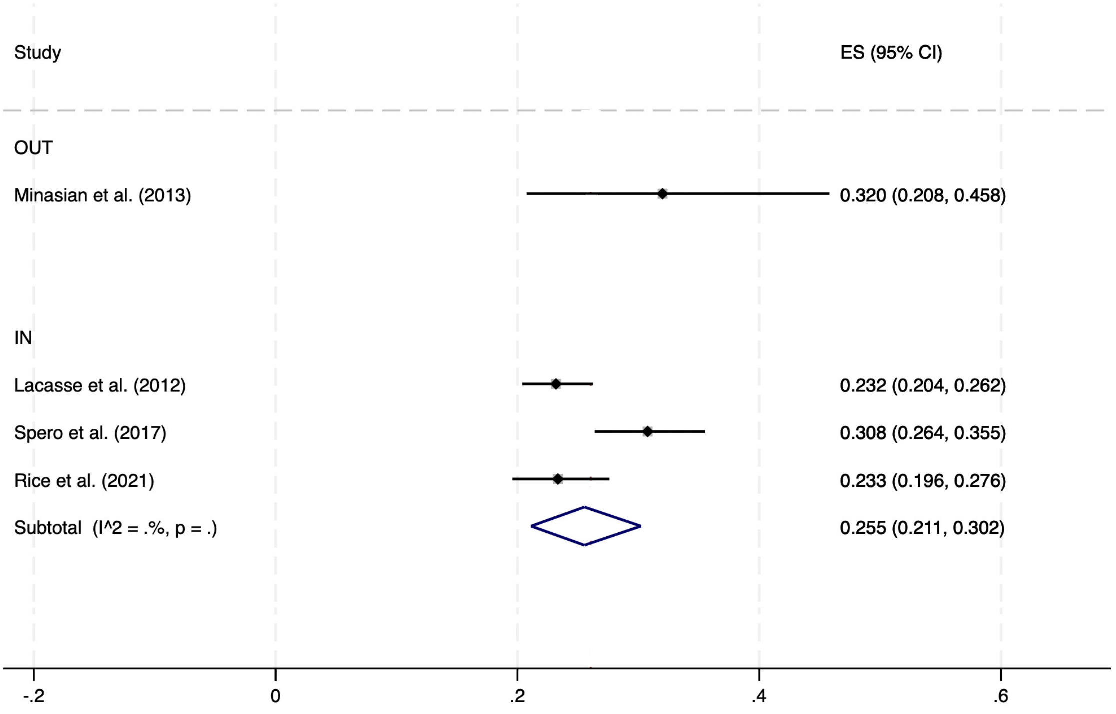

COPD: Clinical diagnosed Chronic Obstructive Pulmonary Disease. CI: Confidence Interval. I^2: level of heterogeneity.
